# Fluorine-19 MRI for detection and quantification of immune cell therapy for cancer

**DOI:** 10.1186/s40425-018-0416-9

**Published:** 2018-10-11

**Authors:** Fanny Chapelin, Christian M Capitini, Eric T Ahrens

**Affiliations:** 1 Department of Bioengineering, University of California San Diego, 2880 Torrey Pines Scenic Drive, La Jolla, CA 92037 USA; 2 Department of Pediatrics and Carbone Cancer Center, University of Wisconsin School of Medicine and Public Health, 1111 Highland Avenue, Madison, WI 53705 USA; 3 Department of Radiology, University of California of San Diego, 9500 Gilman Dr. #0695, La Jolla, CA 92093-0695 USA

**Keywords:** ^19^F MRI, Fluorine-19, Perfluorocarbon, Immunotherapy, Adoptive cell transfer, Cancer, T cell

## Abstract

Over the past two decades, immune cell therapy has emerged as a potent treatment for multiple cancers, first through groundbreaking leukemia therapy, and more recently, by tackling solid tumors. Developing successful therapeutic strategies using live cells could benefit from the ability to rapidly determine their in vivo biodistribution and persistence. Assaying cell biodistribution is unconventional compared to traditional small molecule drug pharmacokinetic readouts used in the pharmaceutical pipeline, yet this information is critical towards understanding putative therapeutic outcomes and modes of action. Towards this goal, efforts are underway to visualize and quantify immune cell therapy in vivo using advanced magnetic resonance imaging (MRI) techniques. Cell labeling probes based on perfluorocarbon nanoemulsions, paired with fluorine-19 MRI detection, enables background-free quantification of cell localization and survival. Here, we highlight recent preclinical and clinical uses of perfluorocarbon probes and ^19^F MRI for adoptive cell transfer (ACT) studies employing experimental T lymphocytes, NK, PBMC, and dendritic cell therapies. We assess the forward looking potential of this emerging imaging technology to aid discovery and preclinical phases, as well as clinical trials. The limitations and barriers towards widespread adoption of this technology, as well as alternative imaging strategies, are discussed.

## Background

Surgery, chemotherapy and radiotherapy have been used for decades as primary strategies against cancer in patients [1]. However, non-specific toxicities to healthy cells and life threatening side effects from chemotherapy and radiation, as well as drug and radiation cancer cell resistance, have motivated investigators to seek new treatment approaches to improve curative outcomes and quality of life. Immunotherapeutic strategies have emerged as a fourth pillar for cancer treatment, which holds promise for less toxic side effects and durable response rates against residual primary cancers and metastases, even if tumors were previously considered chemorefractory.

Throughout life, the immune system actively prevents neoplastic development through immunosurveillance [2]. The innate immune system, including monocytes, macrophages, dendritic cells (DCs) and natural killer (NK) cells, provide front line protection through cancer cell recognition, lysis, and pro-inflammatory cytokine production [3]. T and B cells, main effectors of the adaptive immune system, mediate antigen-specific responses against cancer and can form long term memory [4]. Nonetheless, cancer cells have evolved mechanisms to evade such surveillance, such as MHC downregulation and cytokine secretion, to create an immunoprivileged microenvironment [5]. Adoptive cell therapy (ACT) aims to counterbalance this effect by providing highly activated effector cells into the body. Early treatments developed by Rosenberg et al.*,* comprised of T cells derived from the tumor-bearing host, are referred to as tumor-infiltrating lymphocytes (TILs) [6]. Subsequently, complex in vitro engineering of the T cell receptor (TCR) by gene transfer, as well as de novo MHC-independent targets called Chimeric Antigen Receptors (CAR) were developed [7]. Progress in the design of CARs included optimization of antigen specificities, T cell activation mechanisms, effector function and T cell persistence [8]. Over 300 clinical trials are currently investigating TILs, TCR and CAR T cell therapies [9].

Inherent in the mind’s eye of clinical investigators is that cell trafficking behavior in vivo may be predictive of therapeutic outcomes. For example, in CAR T cell trials against solid tumors [10], basic assumptions are that therapeutic cell survival and trafficking to the tumor sites are required for a putative therapeutic effect. Clinicians are currently blinded as to whether cells reach their desired tissue targets. Effector cell proliferation and enzyme production is another avenue for assaying ACT activity [11]. Overall, surrogate biomarkers capable of visualizing and quantifying sites harboring cells in vivo*,* as well as survival of ACT at tumor and lymphoid organs, would be invaluable for predicting therapeutic response following administration. Indeed the Food and Drug Administration (FDA) is interested in expanding non-invasive imaging platforms of tracking cells to aid in safety monitoring [12]. In 2008, the Cell, Tissues and Gene Therapies Advisory Committee of the FDA Center for Biologics Evaluation and Research stated that sponsors should be encouraged to develop real-time imaging/labeling methods for tracking cells [13]. Non-invasive clinical imaging techniques including Magnetic Resonance Imaging (MRI) and nuclear imaging are candidates for developing real-time, quantitative biomarkers for ACT [14, 15].

In 2010, the FDA’s Center for Devices and Radiological Health started an initiative to reduce unnecessary radiation exposure from medical imaging [16]. MRI can provide anatomical and disease diagnostic information with intrinsic soft tissue contrast without ionizing radiation. Shortly after the invention of proton MRI, the feasibility of fluorine-19 (^19^F) MRI was demonstrated in 1977 by Holland et al. [17]. ^19^F is a natural halogen, non-radioactive isotope of fluorine. ^19^F has a relative sensitivity of 83% compared to ^1^H and essentially devoid in biological tissues of interest [18], providing background-free imaging of ^19^F-based probes. A description of ^19^F MRI physics can be found elsewhere [19]. Fluorine-dense perfluorocarbon (PFC) nanoemulsions have been specifically engineered to be endocytosed, even by non-phagocytic cells in culture [20]. After cell inoculation, ^19^F MRI signal intensity is linearly proportional to ^19^F-atom concentration, enabling unbiased measurements of apparent cell numbers from images [21].

Here, we provide a brief overview of current and emerging experimental strategies to detect ACT using ^19^F MRI. We focus on the characterization of ACT immune cell populations labeled with PFC nanoemulsions including T cells, NK cells and DC vaccines. We describe how this approach can benefit the discovery and preclinical phases of the therapeutic development and potentially clinical trials.

### PFC-based nanoemulsion probes

PFC molecules have properties that are attractive for cell labeling and ^19^F MRI tracking applications [22]. Their strong C-F covalent bonds render them chemically inert and are not metabolized in vivo [23]. Moreover, PFCs often display simultaneous lipo- and hydro-phobic properties [24] and do not dissolve in cell membranes. PFCs commonly used for ^19^F MRI imaging include perfluoropolyether (PFPE), perfluoro-15-crown-5-ether (PCE) and perfluorooctyl bromide (PFOB) [22]. PFPE and PCE are linear and cyclic polymers, respectively, each with numerous chemically-equivalent fluorine yielding high MRI sensitivity. PFOB has less MRI sensitivity overall due to chemically inequivalent F-sites [25].

Neat PFC materials are dense oils. Emulsification is used to make a colloidal suspension of the PFC oil that is stabilized using a surfactant. The surfactant coat can also impart desirable surface properties that promote cell uptake in culture [26, 27]. The most commonly used classes of surfactants are pluronics and phospholipids [28]. Key design considerations in nanoemulsion formulation include a small droplet size (typically 100–200 nm), a narrow size range (e.g., polydispersity index < 0.2) and a high fluorine concentration (~ 20–30% *v*/v) to minimize volume added to culture. Nanoemulsion formulations may also be complexed with fluorophores, for example near infrared dyes, to create ‘dual-mode’ agents [20, 22, 29]. Recent reviews exhaustively cover PFC nanoemulsion design [22, 30].

Different published studies use a range of emulsion particle sizes [20, 31]. The mean emulsion droplet size can impact the cell labeling process [32]. Larger oil droplets (> 200 nm) are effective in labeling flask-adherent cells, such as DCs, where successful wash steps can be implemented and can potentially result in higher overall labeling levels [31]. However, a smaller droplet size (< 180 nm) allows excess agent not taken up by suspended cells, such as lymphocytes, to be discarded with the supernatant during wash. Emulsion production ideally yields a homogenous size distribution, which is easier to achieve with smaller droplet sizes. Unintended, outlying large droplets (‘stability demons’) may evade detection in dynamic light scattering particle size measurements of the batches. These demons can lead to emulsion instability over time [33] and may spin-down with the cells. Overall, in properly designed experiments, free residual emulsion in the cell inoculant is *de minimis* and inconsequential in view of detection limits of the MRI technique.

### Immune cell labeling

Cell labeling in culture is generally performed by simple co-incubation with PFC as another factor in the media, followed by a wash step. Labeling periods range from several hours [21, 34–36] to a day or more [37–39] to allow for endocytic uptake to occur. Determinants of obtainable PFC cell uptake include (i) dose of PFC in media, (ii) cell cytoplasmic volume and (iii) phagocytic properties of cells. Typically, several concentrations and incubation times are tested to optimize uptake while minimizing potential cell viability and phenotype alterations [20].

Lymphocyte labeling can be challenging due to their small cellular and cytoplasmic size that limits the number of nanoemulsion droplets it can hold. In addition, lymphocytes are not naturally phagocytic. Optimal labeling efficiency is attained when cells are in log phase of division. PFC uptake will follow a dose response in the shape of a sigmoidal curve [39]. A critical factor for strong labeling of lymphocytes is that the culture must be viable and actively expanding, typically aided by aggressive cytokine and co-stimulatory molecule engagement (e.g., irradiated 4-1BBL/IL-15 expressing feeder cells, CD3/CD28 beads, etc.) as discussed elsewhere [37, 40]. Preferred PFC nanoemulsion formulations enable labeling of lymphocytes for in vivo tracking without the use of transfection agents [20], as shown in preclinical studies [22, 41] (Table 1). In contrast, macrophages and immature DCs possess a larger cytoplasmic volume and are aggressively phagocytic [42] and thus are more readily labeled to higher levels.

**Table 1:** Overview of ^19^F MRI applications in cell therapy for cancer. SC = subcutaneously, LN = lymph node, CNS = central nervous system, * = clinical trial

Cell type	Recipient species	model	Tracer agent	Therapy delivery route	Imaging post-transfer (day)	Key findings	Reference
T-cell therapy							
Primary BALB/c mouse T-cells	BALB/c mouse	N/A	BODIPy-TR PFPE	Intraperitoneal	2	T cell homing to the abdominal LN	[22]
Human CAR T against EGFRvIII	SCID mouse	glioblastoma SC	CS-1000 ATM	Intravenously	2, 7, 14	CAR T cell homing to the tumors and spleen, reduced tumor growth	[40]
DO11.10 mouse T cells	BALB/c mouse	chicken ova SC	PFPE/PFPE –Alexa657	Intraperitoneal	4, 7, 11, 21	T cell homing to the draining inguinal LN and persistence over 3 weeks	[35]
Naïve T cells, OT-1 T cells	C57BL/6 mouse	melanoma expressing ova	CS-1000 ATM	Intravenously	1	No signal found in the tumor, but found in the chest (lungs), abdomen (liver), and left flank (spleen)	[41]
Pmel-1 cytotoxic T cells	C57BL/6 mouse	CNS glioma	PCE	Intravenously	3, 5, 7, 12	Significant pO2 increase in Pmel-1 treated mice at day 5 compared to controls	[71]
NK cell therapy							
Human NK cells	NSG mouse	neuroblastoma SC	CS-1000 ATM	Subcutaneously or intratumorally	1, 3, 7/8, 10, 15	NK cell detection and persistence at injection sites, no evidence of migration	[37]
Human NK cells	NSG mouse	meduloblastoma CNS	CS-ATM DM Green	Intratumorally	0	In vivo visualization of NK cells after transfer	[36]
DC vaccines							
Mouse bone marrow-derived DC	C57BL/6 mouse	N/A	PCE	Intradermally in the limb	1	Migration of antigen-loaded DC from the footpad to the draining lymph node	[32]
In situ DC labeling	C57BL/6 mouse	CNS glioma	Rhodamine-PCE	Intravenously	1	Migration if In situ labeled DC to CNS tumors, reduced tumor growth	[59]
Mouse bone marrow-derived DC	C57BL/6 mouse	N/A	Rhodamine-PCE	Intradermally in the limb	0	Migration of Erk−/− DC to the draining popliteal lymph node	[59]
Autologous human DC vaccine*	Human	Metastatic colorectal cancer	CS-1000 (PFPE)	Intradermally in quadriceps	0, 1	Succesful first-in-man detection of DC vaccine in patients	[14]
PBMCs							
Human PBMC	Nude mouse, ham shank	N/A	CS-1000 ATM	Intradermally and Intramuscularly	0, 2	Clinical protocol implementation for detection of PBMC in skin and muscle at 1.2 cm depth	[61]

After washes, cell labeling levels can be measured in a pellet sample using conventional ^19^F nuclear magnetic resonance (NMR) spectroscopy to yield the mean ^19^F/cell. Various cell microscopy methods have been used to validate intracellular compartmentalization of PFC droplets. Using transmission electron microscopy, the emulsion droplets appear as electron-sparse ovoids against counterstain [31, 43, 44]. Emulsion droplets often coalesce into encapsulated vesicles consistent with lysosomal storage in lymphoid-type and stem cells [45]. In the case of antigen presenting cells (APCs, e.g., DCs), PFC traffics to more specialized compartments, such as macropinosomes [43].

**Fig. 1:**
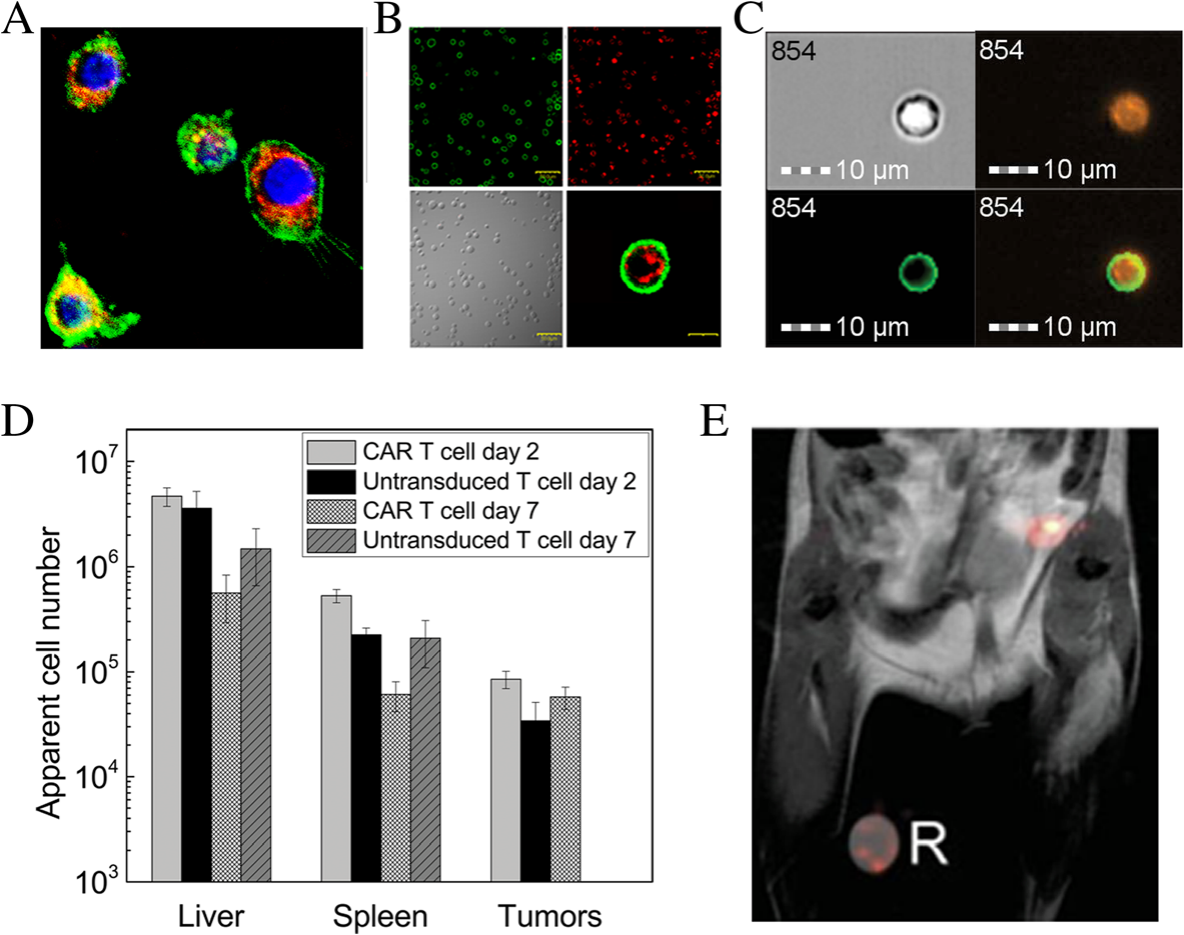
Immune cells labeled with PFC and in vivo distribution. **a** Murine DCs labeled with dual-mode BODIPY-^19^F PFC nanoemulsion as seen in fluorescent micrographs of the cytoplasm (red), along with Hoechst labeled nuclei (blue) and the CD45-FITC labeled cell surface (green). **b** Murine primary activated T cells labeled with dual-mode PFC nanoemulsion showing cytoplasmic localization of CD4-FITC labeled cell surface (green, upper left), the PFC nanoemulsion (red, upper right), white-light image of labeled T cells (lower left) and fusion image of CD4-FITC-PFC (lower right). Scale bar is 20 μm. **c** NK cells isolated from a Balb/c spleen and incubated with a dual-mode PFC agent (BODIPY-^19^F) for 24 h, then incubated with CFSE for 15 min. Upper left: Darkfield microscopy of a Balb/c NK cell. Upper right: BODIPY-^19^F (orange) is seen in the entire cell. Lower left: CFSE (green) is taken up in the cell membrane. Lower right: Fusion image showing labeling with BODIPY-^19^F and CFSE. Scale bar is 10 mm. **d** Biodistribution quantification of fixed tissue samples by ^19^F NMR 2 or 7 days after human CAR T cell treatment in subcutaneous glioma (U87-EGFRvIII) bearing SCID mice. **e** ^1^H/^19^F overlay MRI showing PFPE-labeled antigen specific T cells in the draining lymph node of a BALB/c mouse locally injected with chicken ova. R indicates a reference capillary used for quantification. (Figure adapted from References [22, 35, 40])

Dual-mode, PFC-fluorescence nanoemulsions [20] enable flow cytometry of labeled cells, as well as optical microscopy in histology sections. Confocal microscopy images of labeled immune cells clearly show intracellular localization (Figs. 1a-b). PFC localization is inconsistent with dominate cell surface labeling, which has been confirmed by explicit cell membrane staining (Fig. 1a-b) and by cellular proliferation dyes such as 5(6)-Carboxyfluorescein N-hydroxysuccinimidyl ester (CFSE, Fig. 1c). Detailed fluorescent microscopy studies using a dual-mode emulsion with a pH sensitive dye confirmed that the PFC emulsion traffics into low-pH (lysosomal) vesicles over time [45]. This intracellular compartmentalization is the steady-state in living cells, as the PFC is not degraded in the cell and there is no evidence for active exocytosis [45].

Cell labeling should not alter cell viability, proliferation, phenotypic markers, or function, as described in several reports [46, 47]. In a recent study, Chapelin et al. performed in vitro studies in human CAR T cells showing that PFC labeling does not alter cell viability, division rate and phenotype (defined by CD4/CD8 expression) for at least 14 days post-labeling. Similarly, NK cells labeled with PFPE nanoemulsion exhibited unaltered viability and phenotype [37]. Somanchi et al. published a detailed protocol for expansion and PFPE labeling of NK cells [36]. Cytotoxicity of labeled NK cells against cancer cells in vitro was comparable to non-labeled cells, and cytokine and perforin secretion was preserved [36, 37] (Table 1). The most detailed in vitro study to date involved PFC-labeled primary human DCs [39]; cells were assayed for viability, maturation phenotype, cytokine production, T cell stimulatory capacity, and chemotaxis [39], and no differences in these parameters were observed between labeled and unlabeled cells [39].

### T cells

Adoptive T cell therapy can elicit sustained tumor-specific killing in vivo and has the potential to form long-term memory against tumor-associated antigens. Fundamental questions remain to be answered regarding T cell biodistribution, anti-cancer activity and persistence after infusion. First, non-invasive cell tracking methods could assist in optimizing delivery method (systemic versus local) and dosage. ACT homing to solid tumors remains a challenge, and tracking methods could further our understanding of the factors affecting tumor homing, which may be predictive of response to therapy [48, 49]. Additionally, evaluation of the impact of co-therapies, such as checkpoint inhibitors, by ^19^F MRI could yield insights into the role of adjuvant treatments on T cell behavior.

In preclinical studies, after infusion of PFC-labeled immune cells, one approach for quantitative biodistribution assessment is via conventional ^19^F NMR spectroscopy of intact, fixed tissues samples (i.e., NMR cytometry) [40, 50]. NMR cytometry has the advantage of rapid sample throughput with sensitivity limits of detection of order 10^3^ T cells per sample [40]. In a recent NMR cytometry study, CAR T cells targeting glioma tumors expressing EGFRvIII [40] (Table 1) were labeled with PFC emulsion overnight and subsequently injected IV. Panel necropsy at several time points post-infusion followed by ^19^F NMR measurement of organ fluorine content yielded the apparent transferred cell number in each tissue (Fig. 1d). On average, twice as many CAR T-cells homed to the tumor and spleen compared to naïve T cells. In addition, CAR T cell persistence surpassed that of naïve T cells [40]. Cell quantification in this study did not account for T cell division in vivo. The CAR T cell treatment resulted in significant tumor growth decline and correlated to the number of cells homing to the tumor and spleen.

T cell distribution can also be monitored by ^19^F MRI in vivo cytometry. In early studies, Srinivas et al. [35] labeled antigen-specific DO11.10 mouse T cells with PFC emulsion and infused them into a BALB/c host receiving a local injection of ovalbumin with adjuvant [35] (Table 1). The study tracked the dynamic accumulation and clearance of labeled T cells in the lymph node proximal to the antigen injection site (Fig. 1e). ^19^F MRI allowed for T cell imaging and quantification up to 3 weeks post-transfer. Gonzales et al. [41] used a similar approach in a mouse B16 Ova melanoma tumor model (Table 1). The melanoma cell line was engineered to express Ova and tested using infused PFC-labeled splenocytes, naïve T cells and Ova-peptide activated T cells in vivo. ^19^F MRI images displayed bright hot-spots corresponding to splenocyte and T cell distribution to the lungs, liver and spleen; no cells were detected in tumor by MRI, but could be detected in small numbers by flow cytometry. These results corroborate NMR studies [40] (Table 1).

### NK cells

Another ACT strategy involves infusing NK cells, which are key effectors of innate immunity and by definition not antigen specific. NK cells contribute to cancer immuno-surveillance. They screen local cells in situ and recognize cancer cells expressing altered MHC molecules or downregulated MHC expression, or antibody-coated tumor cells, leading to NK cell perforin release and cancer cell death [51]. Similarly to T cells, NK cell therapies are usually administered intravenously, but also intratumorally [52, 53]. Because NK cells cannot form memory, knowledge of NK cell activity and persistence will be critical to better understand the need for repeated infusions and to develop ‘smarter’ cell delivery methods for solid tumors.

**Fig. 2:**
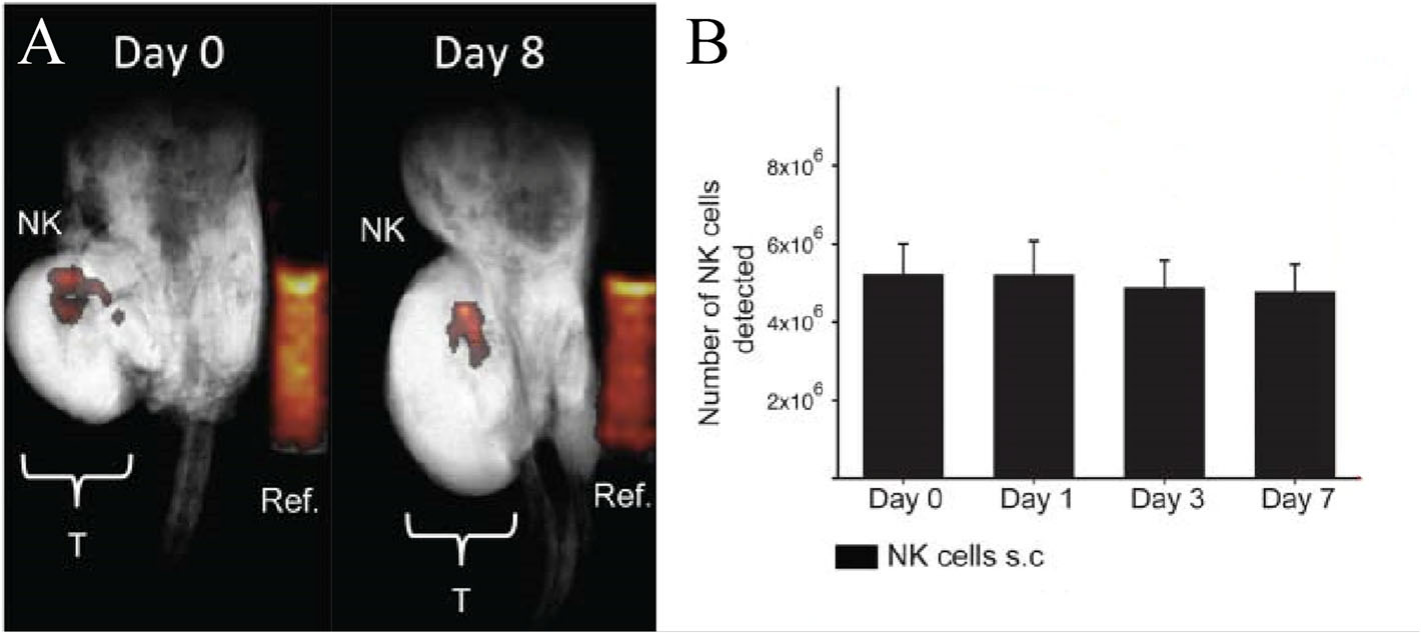
NK cells in mice. **a** In vivo composite ^1^H/^19^F MRI images of ^19^F-labeled human NK cells at day 0 and day 8 post NK therapy in NSG mice bearing human xenograft tumors (Ref. is external quantification reference tube, and “T” is tumor). **b** Mean number of NK cells detected at the tumor site is denoted for each imaging time point. The number of NK cells is stable over a week. (Adapted with permission from Reference [37])

Bouchlaka et al. reported that PFC-labeled human NK cells were detectable by longitudinal MRI up to 8 days after intratumoral injection in NSG mice [37] (Fig. 2a). NK cell number remained relatively stable over 1 week (Fig. 2b). When NKs were injected subcutaneously, NK cell number at the injection site decreased over the same time period and migrated to tumor as evidenced by a reduction in tumor size, although there were too few cells to detect them within the tumor by MRI. NK cells may have insufficient anti-tumor activity and fail to persist in vivo [54]. To palliate such effects, researchers are now incorporating CARs into NK cells, thereby providing antigen-specificity and potentially better anti-tumor activity, with unknown effects on NK persistence [55]. ^19^F MRI may be useful for the development of next generation NK therapies.

### Dendritic cells

In vivo cytometry was originally described and experimentally tested to visualize DCs in mice [43]. DCs are professional APCs that form the link between innate and adaptive immunity. DCs modulate the inflammatory response by precisely activating T cell subtypes such as helper and cytotoxic T cells. DCs are often administered intradermally to facilitate their entry into lymphatic vessels. Therapeutic DCs are usually primed with specific tumor antigens prior to injection to enhance specific antigen presentation and chemokine production [56, 57]. In one study, ‘theranostic’ PFC nanoemulsions were created for one-step DC labeling and tumor priming with antigen [58]. Labeled DCs were injected intradermally, and ^19^F MRI 18 h post-transfer showed DC migration lines toward the draining lymph node [31] (Table 1). In a different study, PFC-labeled mature human DCs were also shown to migrate from a NOD/SCID mouse thigh subcutaneous injection site to the draining popliteal lymph node within 18 h of injection [39]; immature DCs, on the contrary, did not leave the injection site. Ku and coworkers used an in situ cell labeling approach, where PFC nanoemulsion was injected intradermally and taken up by resident DCs, in an effort to visualize DCs migrating into GL261 CNS glioma tumors [59] (Table 1). Injection of rhodamine-conjugated PFC nanoemulsion in either wild type or Erk^−/−^ C57BL/6 mice showed greater fluorine labeled DCs migrating into tumor tissue of Erk^−/−^ C57BL/6 mice and as a result, slower tumor growth. When labeled ex vivo with the same PFC agent, Erk^−/−^ DCs injected intradermally were shown to migrate further towards the popliteal lymph node compared to wild type DCs by ^19^F MRI. Ex vivo ^19^F NMR cytometry of excised lymph nodes quantitatively correlated to the MRI findings. Fluorine labeling may therefore help elucidate regulators of DC migration and enable optimization of DC vaccine therapies.

### Peripheral blood mononuclear cells

**Fig. 3:**
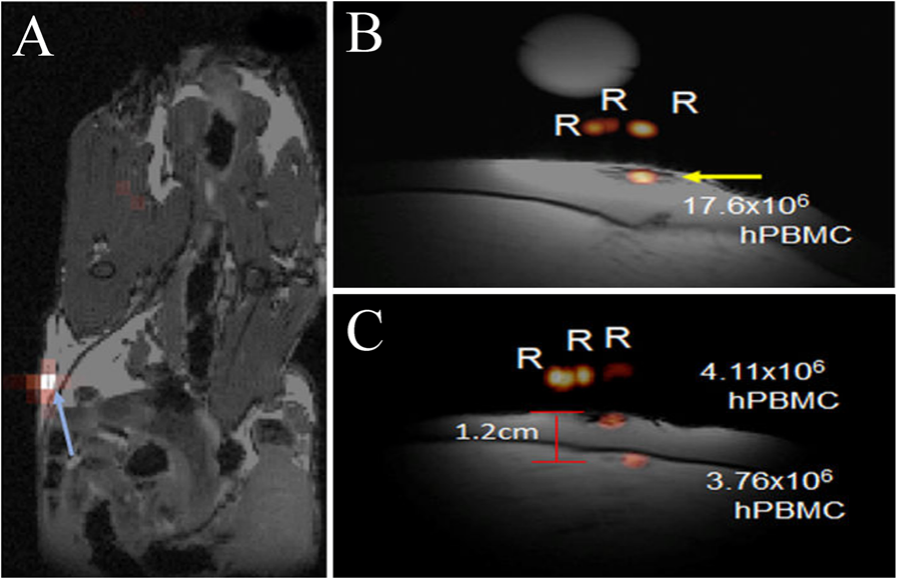
PBMC ^19^F MRI imaging in immunocompromized mice and phantoms. **a** In vivo composite ^1^H/^19^F MRI image of PFPE-labeled human PBMC following subcutaneous flank injection of 6 × 10^6^ cells (blue arrow) in nude mouse. For preliminary clinical MRI protocol implementation, PFPE-labeled PBMC were injected intradermally and intramuscularly in a ham shank phantom. **b** Intradermal injection alone consisted of 20 × 10^6^ cells (yellow arrow). **c** Composite images of shanks receiving both intradermal and intramuscular PBMC injections of 4.5 × 10^6^ cells each. R indicate references used for quantification. (Adapted from Reference [61])

PBMC vaccines encompass both effector cells, (such as T and NK cells) and professional APCs (B cells, monocytes and DCs). Vaccines prepared from PBMCs are FDA-approved for prostate cancer treatment [60]. Fink et al. [61] investigated the use of PFC agents to label human PBMC samples from patients to enable in vivo detection (Table 1). The authors showed that all PBMC cells label, but to varying degrees, and uptake measurements in sorted cell subtypes yielded a labeling (^19^F/cell) profile. When injected in nude mice flanks, PBMC could be detected by ^19^F MRI 2 hours and 2 days post-injection (Fig. 3a). To optimize clinical ^19^F MRI protocols for PBMC vaccine imaging in patients, the authors injected PFC-labeled human PBMC in ham shanks. Both intradermal (Fig. 3b) and intramuscular (Fig. 3c) PBMC injections were detected by clinical 3 T MRI using a custom surface coil at high sensitivity with a detection limit of ~ 6 × 10^4^ PBMC.

### Intracellular oximetry as a biomarker for cancer immunotherapy

**Fig. 4:**
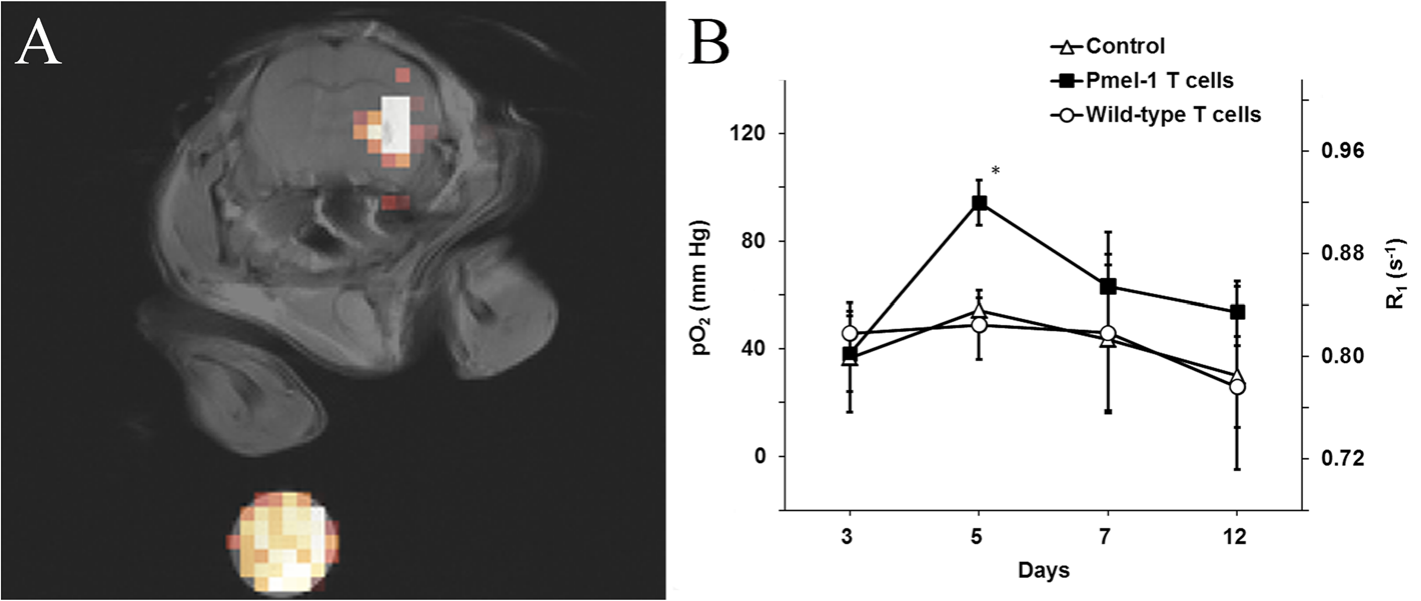
Indirect visualization of T cell therapy efficacy via cancer cell oximetry. **a** Composite ^19^F and ^1^H image of PCE labeled glioma (GL261) cells in the right striatum 5 days after tumor inoculation in C57BL/6 mice. A diluted PCE reference capillary is placed below the animal (bottom). **b** In vivo longitudinal tumor pO_2_ measurement after Pmel-1 mouse derived CD8+ T cell, wild-type T cell injection or no treatment. Transient hyperoxia is observed with administration of Pmel-1 CD8+ T cells. (Adapted from Reference [71])

An intrinsic property of PFCs is that they display weak molecular cohesion, enabling gas dissolution [24]. In fact, extensive work was conducted in the late 1990’s [62, 63] to emulsify PFCs into biocompatible, excretable, and readily injectable blood substitutes to address hospital blood shortages [64]. Building on in vivo cytometry technology, a logical extension is to exploit known bio-sensing properties of the PFC molecules inside the cell. Specifically, certain PFC molecules readily coordinate paramagnetic oxygen, which shortens the ^19^F spin-lattice relaxation time (T_1_), where T_1_ varies linearly with the absolute partial pressure of oxygen (pO_2_) [65]. (T_1_ is the characteristic time constant for the ^19^F nuclei to align along the MRI’s magnetic field, on the order of 0.5 to 2 s.) PFC emulsions have previously been used to measure pO_2_ in vivo using MR techniques [66–69]. However, a novel use of ^19^F-based cell tracking is to use ^19^F T_1_ measurements to monitor intracellular oximetry. The first study using in vivo cytometry to investigate cancer cell pO_2_ changes in response to therapy was performed in a 9 L rat model of brain glioma [70]. Authors showed that treatment with chemotherapy (BCNU) induced a significant and sustained pO_2_ increase in the labeled cancer cells. A follow-up study used a similar approach to monitor intracellular oxygen changes of murine GL261 glioma cells in response to Pmel-1 cytotoxic T cells [71] (Table 1). Labeled glioma cells appear as a background-free hotspot overlaid on a proton image (Fig. 4a). A voxel (volume element) encompassing the hotspot is delineated, and MRI spectroscopy methods yield the voxel R_1_ = 1/T_1_ (Fig. 4b); absolute pO_2_ is then calculated from a calibration curve. MRI results correlated to histopathology analysis, confirming small numbers (~ 10^3^) of infiltrating cytotoxic T cells in the tumor region. These studies demonstrate the feasibility of using in vivo cytometry for real-time, cell-specific oximetry as an early biomarker of anti-cancer responses before MRI-visible tumor shrinkage is observed.

### Limitations of PFC labeled cells

Generally, with PFC labeled cells having a mitotic phenotype, cell division and subsequent dilution of the intracellular label can potentially limit long-term studies and decrease the accuracy of cell quantification [40]. There is no evidence for active exocytosis or degradation of the PFC droplets once internalized by viable cells. Death of labeled cells leads to dispersion of the reagent and thus a loss of ^19^F signal. Potentially, the PFC droplets can also be transferred to macrophages that have engulfed dead cells; if a large number of these macrophages remain in a region of interest, quantification accuracy may suffer. Importantly, the ^19^F signal values clearly diminish at cell injection sites over time if the cells are apoptotic, and this cell loss is accurately quantifiable in longitudinal scans [14, 72], which is an advantage over prior-art iron-oxide nanoparticle based cell tracking approaches [73, 74]. Ultimately, clearance of PFC agents from the body occurs via uptake by cells of the RES, particularly Kupffer cells of the liver, followed by lung exhalation [75]. In fact, the ^19^F liver signal, and the effective number of cells represented by this value, can be used as a proxy to calculate the dead fraction of the infused cell product [40].

### Cell sensitivity

Since its introduction in clinical practice in the 1980s, MRI has experienced remarkable growth and development. But implementation of new clinical applications comes with challenges both technical and logistical in nature. Often a key limitation of ^19^F MRI probes is sensitivity. Unlike conventional ^1^H MRI, where the probe (water) concentration (> 100 Molar ^1^H) and thus sensitivity is high, ^19^F MRI is limited by the total amount and distribution of fluorine atoms introduced into the subject’s tissue. The limits of detection using ^19^F-based imaging ranges from ~ 10^3^ to ~ 10^5^ cells per voxel [76]. For a given experiment, results depend on specific details, such as the PFC molecule and nanoemulsion used, the cell type (i.e., cell cytoplasm size) labeled, viability of cell culture and commensurate label uptake, image acquisition methods, magnetic field strength, and MRI detector configuration [40, 46, 61, 72]. Looking forward, there are multiple, clinically-feasible, technical avenues for improving cell detection sensitivity that are vigorously being investigated involving new probe design and data acquisition methods [30, 77, 78].

### Future clinical perspective

**Fig. 5:**
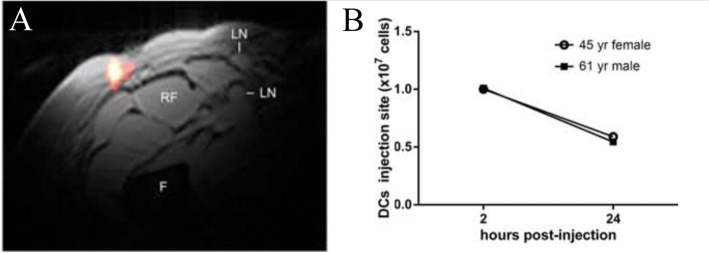
Clinical DC vaccine imaging following intradermal administration in patients with colorectal cancer. **a** In vivo composite ^1^H/^19^F MRI image of (10^7^) PFPE-labeled autologous DCs 4 h after intradermal injection in a 53-year-old female patient (F = femur, RF = rectus femoris, LN = inguinal lymph node). **b** Quantification of apparent DC numbers using the in vivo ^19^F MRI data, measured in two patients. At 24 h post-inoculation, half of the injected DCs are detected at the injection site. (Adapted from Reference [14])

^19^F MRI cell detection techniques are just beginning to be employed in clinical trials (Table 1), and feasibility has been established in a first-in-human clinical study [14]. An autologous DC vaccine was labeled with a PFC nanoemulsion ex vivo and re-injected into colorectal cancer patients intradermally (Fig. 5a). ^19^F MRI enabled visualization of injected DCs at the injection site and longitudinal persistence evaluation (Fig. 5b).

When engaging cell therapy regulatory agencies, such as the US FDA, safety is the primary concern. Within the FDA, ^19^F labeled therapeutic cells are considered a combination product and regulated by the Center for Biologics Evaluation and Research (CBER). Generally, PFC is viewed as having a favorable safety profile and is used in several FDA-approved medicines [79], as well as for contrast-enhanced ultrasound [80]. For cellular therapies, the release criteria for PFC labeled cell batches should match the release criteria expected for the unlabeled cell product [14], such as total nucleated cell count, cell viability, Gram stain, bacterial contamination and endotoxin levels.

Post-infusion, cell viability and anti-tumor efficacy of PFC labeled cells may also be examined in preclinical studies as part of the investigational new drug (IND) application for the cell therapy product. However, imaging results in rodent models of cellular immunotherapy can have significant limitations and may not well reflect how the cell product will behave in patients. Besides the obvious immunological dissimilarities, particularly with immunodeficient xenograft models, typical total cell number doses infused in ACT trials are vastly higher in human trials compared to mice (~ 10^10^ versus 10^6^, respectively). Dosing on a cell number/kg basis can help predict translation to clinical dosing. However, because tumor size may be of similar order of magnitude in size in rodent and humans, scaling the absolute number of therapeutic cells homing to patients’ tumors may be difficult to predict.

As experience with PFC labeling of cell therapy products grows, additional considerations may also be needed, for example, in the clinical batch scale-up of the labeling process [81] in specialized facilities. Furthermore, one could imagine having a cell therapy product expanded at a third-party site with a PFC label incorporated, and then shipped as a refrigerated or cryopreserved pre-labeled cell product; similar workflows are already in place for unlabeled, FDA-approved DC and CAR T cell products for cancer patients. Our view is that routine labeling of large cell batches can be engineered into a well-controlled process that can be exportable to multi-site clinical trials.

Additional logistical limitations to the development of routine fluorine imaging include the fact that clinical scanners are most often equipped for proton scans only. ^19^F MRI requires specialized detection coils and hardware modifications for image acquisition, which are not currently available in most MRI centers, but can be sourced by third parties [82, 83].

### Alternative cell detection strategies – Nuclear imaging

The potential use of radionuclide-based imaging methods, particularly PET and SPECT, are an alternative to ^19^F MRI cell detection [15, 84]. Generally, nuclear imaging methods have a high potential sensitivity in vivo*.* Detection of cells labeled with radioactive tracers ex vivo is feasible, but can be challenged by passive leakage of the radioactive tracer from labeled cells, potential radiotoxicity to cells, and a limited time window for scanning due to the limited half-life of the radioisotope. The use of radiolabeled leukocytes has precedent clinically for diagnostic inflammation detection. For example, Ceretec™ (GE Healthcare), a SPECT labeling agent containing radioactive technetium-99 to label white cells ex vivo that are reinfused, is an FDA-approved diagnostic for intra-abdominal infection and inflammatory bowel disease.

Other nuclear imaging approaches employ gene reporters [85, 86]. Reporters require vector transduction of therapeutic cells prior to infusion. Subsequently, a radioactive substrate is infused systemically in vivo to enable imaging of transduced cells. This approach has the benefit of the potential for long-term detection of cell products that proliferate in vivo. Current PET tracers with potential for clinical cell therapy imaging include HSV-FIAU [87] and [^18^F] F-Ara-G [88] reporters. Reporters require high-efficiency cell transduction manipulations and would not be practical for certain autologous cells like TILs. The ^18^F has a half-life of ~ 110 min thereby limiting longitudinal studies from a single substrate dose.

Another alternative is PET diabody technology that uses antibody fragments against CD4 and CD8 receptors with ^89^Zr or ^64^Cu (half-lives 768 and 13 h, respectively) resulting in specific targeting of T-cells in vivo [89, 90]. This technology does not require ex vivo manipulation of the cells but does not distinguish between endogenous host cells and adoptively transferred cells in vivo [91]. Overall, cell quantification in situ using PET reporter and antibody-based approaches present several challenges to date but remain an emerging area of research.

## Conclusion

Our view is that cell labeling is a well-controlled and validated process that has been reproduced by numerous laboratories. The properties of labeled cells, such as labeling levels (i.e., mean ^19^F/cell) and intracellular localization of PFC, are predictable based on intrinsic phagocytic tendencies, physical cell size, high-level function in the body, and cell activation status and health during the labeling process. Fluorine MRI enables noninvasive monitoring of in vivo survival and behavior of therapeutic cells, as well as their indirect effect on cancer cells. Overall, the use of ^19^F-based MRI cell detection of cell therapy products in vivo is still in the early adaptor phase, but holds promise for advancing a wide range of cell therapy trials for cancer.

## Abbreviations

ACT: Adoptive cell therapy
CAR: Chimeric antigen receptor
CFSE: 5(6)-Carboxyfluorescein N-hydroxysuccinimidyl ester
DC: Dendritic cell
EGFRvIII: Epidermal growth factor receptor variant three
MHC: Major histocompatibility complex
MRI: Magnetic resonance imaging
NK: Natural killer
NMR: Nuclear magnetic resonance
PBMC: Peripheral blood mononuclear cells
PCE: Perfluoro-15-crown-5-ether
PET: Positron emission tomography
PFC: Perfluorocarbon
PFOB: Perfluorooctyl bromide
PFPE: Perfluoropolyether
SPECT: Single photon emission coherent tomography
TCR: T cell receptor
TIL: Tumor infiltrating lymphocyte
